# Whole-Genome Sequence of a *Pantoea* sp. Strain Isolated from an Olive (Olea europaea L.) Knot

**DOI:** 10.1128/MRA.00978-19

**Published:** 2019-10-17

**Authors:** Gabriela Vuletin Selak, Marina Raboteg, Audrey Dubost, Danis Abrouk, Katja Žanić, Philippe Normand, Petar Pujić

**Affiliations:** a Institute for Adriatic Crops and Karst Reclamation, Split, Croatia; b Ecologie Microbienne, Centre National de la Recherche Scientifique UMR 5557, Université de Lyon, Université Claude Bernard Lyon I, INRA, UMRA1418, Villeurbanne, France; Indiana University, Bloomington

## Abstract

Here, we present the total genome sequence of *Pantoea* sp. strain paga, a plant-associated bacterium isolated from knots present on olive trees grown on the Adriatic Coast. The genome size of *Pantoea* sp. paga is 5.08 Mb, with a G+C content of 54%. The genome contains 4,776 predicted coding DNA sequences (CDSs), including 70 tRNA genes and 1 ribosomal operon. Obtained genome sequence data will provide insight on the physiology, ecology, and evolution of *Pantoea* spp.

## ANNOUNCEMENT

*Pantoea* is a genus of Gram-negative bacteria belonging to phylum *Proteobacteria*, class *Gammaproteobacteria*, order *Enterobacteriales*, family *Erwiniaceae*. In the olive knot disease, the nonpathogenic bacterial species Pantoea agglomerans and Erwinia toletana, olive plant epiphytes and endophytes, coexist with the pathogenic bacterium Pseudomonas savastanoi pv. savastanoi, the causal agent of disease (1–3).

*P. agglomerans* strains inhabiting olive trees as nonpathogenic endophytes have been shown to cooperate with strains of *P. savastanoi*, forming a stable bacterial consortium that increases the severity of disease (1). It has been suggested that the indole-3-acetic acid (IAA) and cytokinins produced by *P. agglomerans* may increase the size of the knots on olive caused by *P. savastanoi* and also that the growth of *P. agglomerans* is apparently supported by the copresence of an actively growing population of *P. savastanoi* (2).

Bacterial cells were isolated from olive knots isolated in the central region of Dalmatia, Croatia (global positioning system [GPS] coordinates 43°30′19.6″N, 16°29′55.0″E). Fresh olive knots were surface sterilized by 75% ethanol, sliced with a sterile scalpel, and plated onto King’s medium agar plates (4). Plates were kept in the dark at 25°C for 48 h. An isolated single colony was transferred into 10 ml of liquid lysogeny broth (LB) medium and grown at 28°C for 24 h with shaking. DNA was isolated from bacterial cells using a microbial DNA kit (Macherey-Nagel, Hoerdt, France).

Two micrograms of genomic DNA was used for fragmentation and 300-bp library preparation using a Nextera XT DNA library prep kit and protocol (Illumina, Evry, France), and 2 × 150-bp paired-end sequencing was done using Illumina technology at GATC Biotech (Mulhouse, France). Quality controls were made with FastQC (5) and Trimmomatic (6). We obtained a total of 7,822,775 reads, with 462× coverage and a G+C content of 54%. Genome assembly was realized using Unicycler v0.4.3 software, and annotation was done with the MicroScope platform v3.10.0 using default parameters, with a minimum contig length of 200 bp (7–9). The *Pantoea* sp. genome is assembled in 52 contigs, with the largest contigs of 1,219,567 bp. The genome size has 5,077,592 bp, an *N*_50_ value of 580,527 bp, a G+C content of 54.58%, and 4,786 annotated genes.

### Data availability.

The complete genome sequence described here has been deposited at NCBI/GenBank under BioProject number PRJNA555164, BioSample number SAMN12302724, and GenBank accession number VLTF00000000. Raw reads were deposited at NCBI under SRA accession number SRX6799334. The version described in this paper is the first version. The *Pantoea* sp. paga strain is available on request from the Institute for Adriatic Crops (IAC).
